# Basic residues at the C-gate of DNA gyrase are involved in DNA supercoiling

**DOI:** 10.1016/j.jbc.2021.101000

**Published:** 2021-07-22

**Authors:** Eric M. Smith, Alfonso Mondragón

**Affiliations:** Department of Molecular Biosciences, Northwestern University, Evanston, Illinois, USA

**Keywords:** structure–function, DNA topoisomerases, mutagenesis, protein structure, protein–DNA interactions, BSA, bovine serum albumin, G-segment, gate segment, T-segment, transport segment, WHD, winged-helix domain

## Abstract

DNA gyrase is a type II topoisomerase that is responsible for maintaining the topological state of bacterial and some archaeal genomes. It uses an ATP-dependent two-gate strand-passage mechanism that is shared among all type II topoisomerases. During this process, DNA gyrase creates a transient break in the DNA, the G-segment, to form a cleavage complex. This allows a second DNA duplex, known as the T-segment, to pass through the broken G-segment. After the broken strand is religated, the T-segment is able to exit out of the enzyme through a gate called the C-gate. Although many steps of the type II topoisomerase mechanism have been studied extensively, many questions remain about how the T-segment ultimately exits out of the C-gate. A recent cryo-EM structure of *Streptococcus pneumoniae* GyrA shows a putative T-segment in close proximity to the C-gate, suggesting that residues in this region may be important for coordinating DNA exit from the enzyme. Here, we show through site-directed mutagenesis and biochemical characterization that three conserved basic residues in the C-gate of DNA gyrase are important for DNA supercoiling activity, but not for ATPase or cleavage activity. Together with the structural information previously published, our data suggest a model in which these residues cluster to form a positively charged region that facilitates T-segment passage into the cavity formed between the DNA gate and C-gate.

DNA topoisomerases are highly conserved enzymes that can alter and maintain the topological state of DNA during cellular processes such as replication and transcription (1, 2, 3). When cellular machinery access DNA by strand separation, positive supercoils, negative supercoils, and catenated DNA strands can be created both in front of and behind the sites of action (2). Topoisomerases are grouped into one of two classes based on the mechanism they use to resolve these topological states. Type I topoisomerases make a single-stranded break in the DNA, whereas type II topoisomerases make a double-stranded break in the DNA. Topoisomerases are further divided into subclasses (type IA, IB, or IC and type IIA, or IIB) by both sequence and structural similarities (1). Both types use either one (type I) or two (type II) highly conserved catalytic tyrosine residues to perform a nucleophilic attack on the phosphodiester backbone of DNA (1). Owing to their essential role in chromosomal maintenance, type II topoisomerases are targets for both anticancer therapeutics and antibiotic compounds (4, 5, 6).

DNA gyrase is a bacterial type IIA topoisomerase that works *via* an ATP-dependent two-gate, strand-passage mechanism. DNA gyrase is an A_2_B_2_ heterotetramer (1, 7) formed by GyrA and GyrB subunits. GyrB contains a conserved ATPase domain, a transducer region, and a metal-binding topoisomerase–primase domain (Fig. 1*A*). GyrA contains a winged-helix domain (WHD), a tower domain, a large coiled-coil region, and a C-terminal domain (Fig. 1*A*). DNA gyrase has the unique ability among type IIA topoisomerases to negatively supercoil DNA, and it carries out this reaction through DNA wrapping around its C-terminal domains (8, 9). DNA gyrase is able to carry out its ATP-dependent strand-passage mechanism by making use of three gates that are formed by the heterotetramer (Fig. S1*A*). First, a segment of ssDNA, called the gate segment (G-segment), binds to a gate, the DNA gate, that is formed by the WHD of GyrA and the topoisomerase–primase domain of GyrB (Fig. S1*A*) (10). Then, DNA gyrase interacts with a second DNA double strand, called the transport segment or T-segment, in the N-gate, which is formed by two ATPase domains, one domain coming from each GyrB monomer (Fig. S1*A*) (11, 12). The N-gate closes upon ATP binding, trapping the T-segment in the cavity formed between the N-gate and DNA gate (13, 14). Next, the G-segment is cleaved and the DNA gate is opened (15). Upon ATP hydrolysis, the T-segment is propelled through the open DNA gate into the cavity formed between the DNA gate and the C gate (16, 17). Finally, the G-segment is religated and the T-segment exits the enzyme through the C-gate that is formed by the coiled-coil domains of the GyrA dimer (Fig. S1*A*) (18).

**Figure 1:**
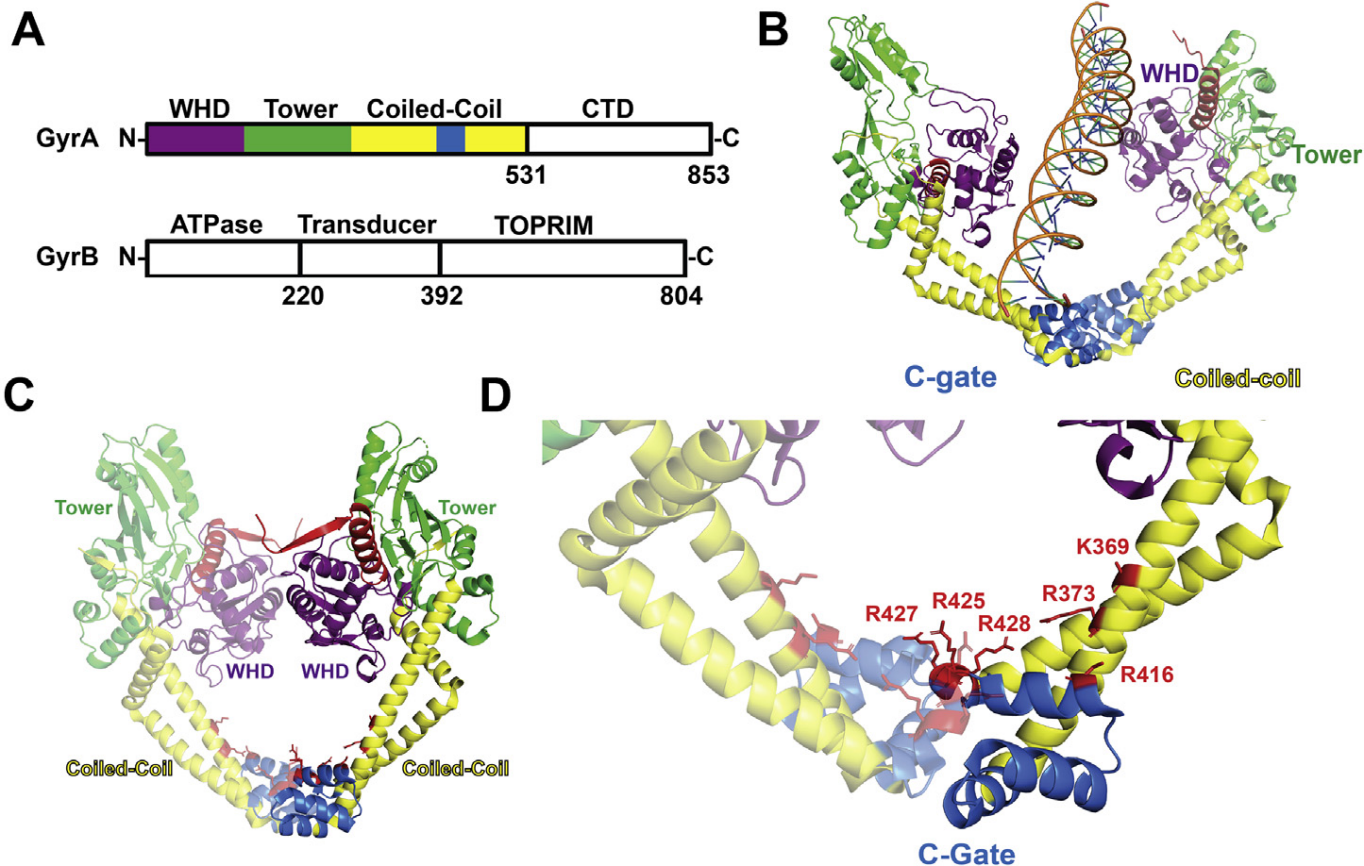
**Structures of GyrA provide insights into T-segment passage.***A*, domain diagrams of GyrA and GyrB. GyrA contains an N-terminal winged-helix domain (WHD), a tower domain, a coiled-coil region, and a C-terminal domain (CTD). GyrB contains an ATPase domain, a transducer, and a topoisomerase–primase (TOPRIM) domain. *B*, *cartoon* representation of a portion of the dihedral oligomeric complex of GyrA in complex with DNA (PDB ID: 6N1P) (20). Two GyrA dimers are shown with an open DNA gate. The DNA is shown in close proximity to the C-gate. Depicted in *green* is the tower domain, the winged-helix domain is depicted in *purple*, and the coiled-coil is shown in *yellow*. The portion of the coiled-coil that makes up the C-gate is depicted in *blue*. *C*, GyrA from the crystal structure of a DNA gyrase–DNA cleavage complex from *S. pneumoniae* (PDB ID: 4Z2C). For clarity, one of the GyrA dimers is presented as a *transparent cartoon*. Domains are colored as in panel *B*. *D*, zoomed-in view of the C-gate from panel *C* depicting the mutated conserved basic residues as *red sticks*.

Although many important details surrounding the strand-passage mechanism of DNA gyrase and other type IIA topoisomerases have been elucidated, there are still missing details about the mechanism, particularly information about how the T-segment passes through the C-gate. Important information about this step was provided by crystal structures from *Saccharomyces cerevisiae* topoisomerase II and from human topoisomerase II (10, 19). Those structures depict the C-gate in an open conformation, providing insights into how C-gate movement allows T-segment exit. However, it was not until the cryo-EM structures of oligomers of *Streptococcus pneumoniae* DNA GyrA were solved that the T-segment was observed passing through the DNA gate and into close proximity of the C-gate (20). Although these structures provided important details about the mechanism of action of type II topoisomerases, additional molecular details about how the T-segment travels into the cavity formed between the DNA gate and the C-gate need to be elucidated. Here, we report a focused alanine scanning mutagenesis screen that identifies basic residues on the C-gate that are important for DNA gyrase supercoiling activity but are dispensable for ATPase or cleavage activity. These data, taken together with the previous cryo-EM structures of DNA gyrase, suggest that a basic patch within the C-gate is involved in mediating T-segment passage through the enzyme.

## Results

### Sequence homology and structure-guided site-directed mutagenesis of GyrA map residues involved in T-segment passage

Numerous structures of type IIA topoisomerases exist from a variety of organisms, ranging from eukaryotes such as *S. cerevisiae* through bacteria, and some archaea, such as *Methanosarcina mazei* (21, 22, 23, 24, 25). Despite the large amount of existing structural information, there are very few depictions of any type II topoisomerase enzyme interacting with a T-segment, most likely because of the predicted transient nature of this interaction. However, a recent cryo-EM structure of *S. pneumoniae* GyrA depicts a putative T-segment in close proximity to the C-gate (20). The *S. pneumoniae* GyrA model shows the T-segment 3 to 12 Å away from the surrounding basic C-gate residues that are pointing into the cavity. Therefore, we hypothesized that these C-gate residues interact with the T-segment and facilitate T-segment passage out of the enzyme (Fig. 1*B*). To identify DNA gyrase residues in the C-gate that could be directly involved in T-segment passage, we first performed sequence alignments between *S. pneumoniae* GyrA and GyrA from other bacteria. This allowed us to identify several highly conserved basic residues in the coiled-coil and globular regions of the C-gate (Fig. S1*B*).

We predicted that those residues which face into the cavity formed between the DNA gate and the C-gate would be poised to interact with a T-segment as it traverses through the enzyme (Fig. 1, *C* and *D*). Upon inspection of the crystal structure of *S. pneumoniae* GyrA, two residues, K369 and R373, that reside along the long helix of the coiled-coil domain facing toward the cavity, were identified (Fig. 1, *C* and *D*) (26). In addition to these residues, four arginine residues (R416, R425, R427, and R428) were found on the portion of the coiled-coil that makes up the C-gate. The side chains of R425, R427, and R428 point directly into the cavity the T-segment must pass through (Fig. 1, *C* and *D*). R425, R427, and R428 are also found near the dimer interface that forms the C-gate, with all six side chains spanning a distance of approximately 9 Å. Therefore, we hypothesized that these residues form a basic patch that may be poised to interact with the T-segment.

### Basic residues in the C-gate are important for DNA gyrase supercoiling activity

**Figure 2:**
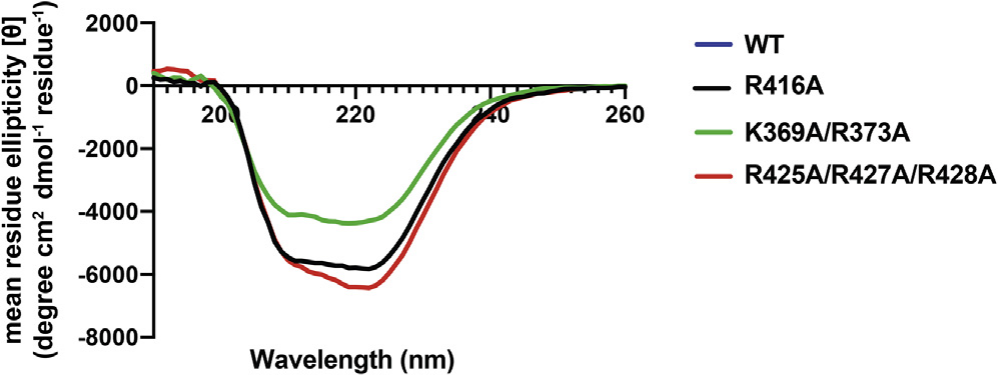
**CD spectra of alanine mutants in GyrA.** CD spectra are shown graphically with the y-axis depicting mean residue ellipticity (degree cm^2^dmol^−1^residue^−1^) and the x-axis showing wavelength in nanometers (nm). The spectrum for the WT GyrA is shown in *blue*, the GyrA–R416A mutation in *black*, GyrA–K369A/R373A mutant in *green*, and the GyrA–R425A/R427A/R428A mutant in *red*. Spectra of all constructs were acquired after dilution of the proteins to 10 μM in 50 mM Tris HCl, pH 8.0, 600 mM KCl, and 1 mM EDTA before being placed in a quartz cuvette with a path length of 0.5 mm.

To determine if the identified conserved residues were important for DNA gyrase supercoiling activity, we introduced three separate sets of mutations into *S. pneumoniae* GyrA (Fig. 1 and Fig. S1, highlighted in red). We engineered an R425A/R427A/R428A triple mutant, a K369A/R373A double mutant, and an R416A single mutant, spanning the three basic regions observed in the structure. Recombinantly expressed and purified WT and mutant GyrA proteins were analyzed by CD to ensure that the introduced mutations did not grossly affect the secondary structure of the protein (Fig. 2 and Fig. S2). Consistent with the structure of *S. pneumoniae* GyrA, CD spectra for WT GyrA suggests a large alpha-helical content. The R416A or R425A/R427A/R428A mutations resulted in similar spectra as WT GyrA, suggesting that mutation of these residues does not grossly affect protein structure (Fig. 2). Residues K369 and R373 lie within an alpha-helix that makes up part of the coiled-coil domain of GyrA. While the CD spectrum for the K369A/R373A double mutant has the same overall shape as WT GyrA, the slightly dampened signal may indicate a partially perturbed secondary structure in this region (Fig. 2). The gel filtration elution profile of this mutant was also subtly different from GyrA containing either the R416A mutation or the R425A/R427A/R428A mutations (Fig. S2).

**Figure 3:**
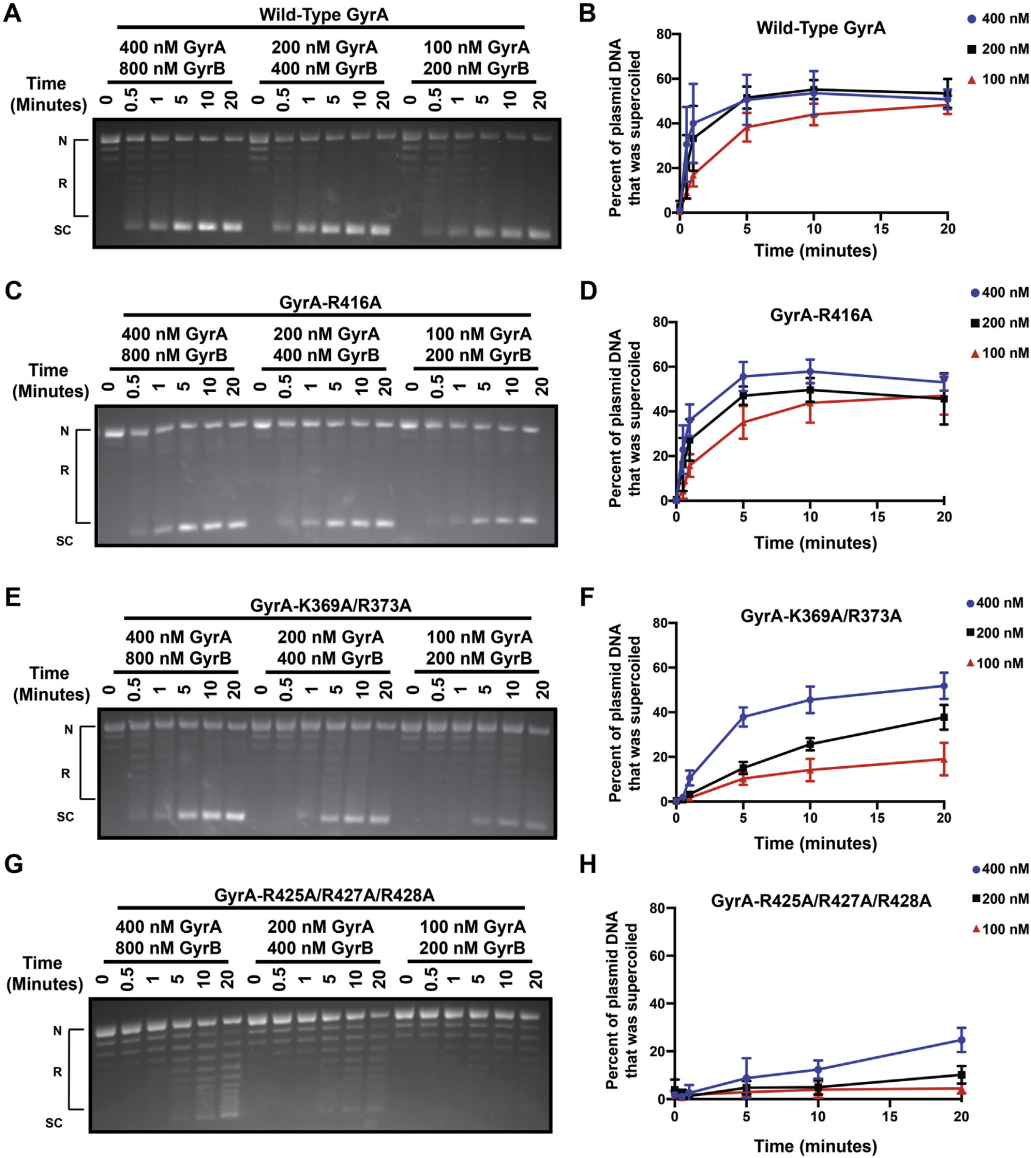
**Supercoiling activity of DNA gyrase containing alanine mutations in GyrA.***A*, *C*, *E* and *G*, representative ethidium bromide–stained 1% agarose gels showing the supercoiling activity of DNA gyrase reconstituted with the indicated construct of GyrA and WT GyrB. The final concentration of each protein component used in each set of reactions is indicated above the lanes they correspond to. 10 nM pBR322 plasmid was used in each reaction. Nicked, relaxed, and supercoiled pBR322 plasmid are indicated by N, R (topoisomers are indicated by *brackets*), and SC, respectively, to the *left* of the gel. The *top band* is a mixed population of fully relaxed plasmid DNA and nicked DNA; thus, the N is aligned with the *top* of the *bracket* in each gel. The zero time point contains all reaction components but was taken before the addition of ATP. The five additional time points are counted after ATP addition to each reaction. *B*, *D*, *F* and *H*, plots showing the percent of plasmid DNA that has been supercoiled on the y-axis and time (minutes) on the x-axis. Gyrase is reconstituted with WT GyrB, and the indicated GyrA construct. The data for reactions carried out with gyrase formed with 400 nM GyrA and 800 nM GyrB are shown in *blue*, 200 nM GyrA and 400 nM GyrB in *black*, and 100 nM GyrA and 200 nM GyrB in *red*. Data points are averaged values from three independent supercoiling experiments, and the error bars denote the SD from the three independent experiments.

We asked if these conserved basic residues are important for DNA gyrase supercoiling activity using time-based supercoiling assays. To detect subtle defects in supercoiling, three different concentrations of DNA gyrase (400 nM GyrA:800 nM GyrB, 200 nM GyrA:400 nM GyrB, and 100 nM GyrA:200 nM GyrB) were used. All concentrations of WT DNA gyrase that were tested efficiently supercoiled the pBR322 plasmid within 20 min (50.8%, 53.4%, and 48.3% of the plasmid was supercoiled in 20 min, respectively) (Fig. 3, *A* and *B* and Fig. S3). DNA gyrase reconstituted with GyrA–R416A exhibited a similar supercoiling activity as WT at all concentrations and time points tested (53%, 45.6%, and 47% of the plasmid was supercoiled in 20 min, respectively) (Fig. 3, *C* and *D*). DNA gyrase reconstituted with GyrA–K369A/R373A displayed no defect in supercoiling activity at the highest concentration but was less active at lower protein concentrations (51.9%, 37.7%, and 19.1% of the plasmid was supercoiled in 20 min) (Fig. 3, *E* and *F*). It is possible that these residues are involved in guiding the T-segment out of the C-gate; however, we cannot rule out the role of potential structural defects imparted on GyrA by introducing these mutations, as suggested by our CD data (Fig. 2). Gyrase harboring the triple mutant, GyrA–R425A/R427A/R428A, had a drastically reduced supercoiling activity with only 25% of the relaxed plasmid DNA being supercoiled in 20 min at the highest enzyme concentration, and a mere 10% and 4% of plasmid DNA being supercoiled in 20 min at the two lower enzyme concentrations (Fig. 3, *G* and *H*). A similar defect was observed when testing the triple mutant alongside WT in supercoiling assays with relaxed pUC19 plasmid DNA (Fig. S3), confirming that the result is not plasmid specific. Together, these data suggest that three conserved residues, R425, R427, and R428, are pivotal for DNA gyrase supercoiling activity, and two additional residues, K369 and R373, may also play a role in DNA supercoiling.

### Gyrase-harboring mutations in the C-gate can cleave DNA

**Figure 4:**
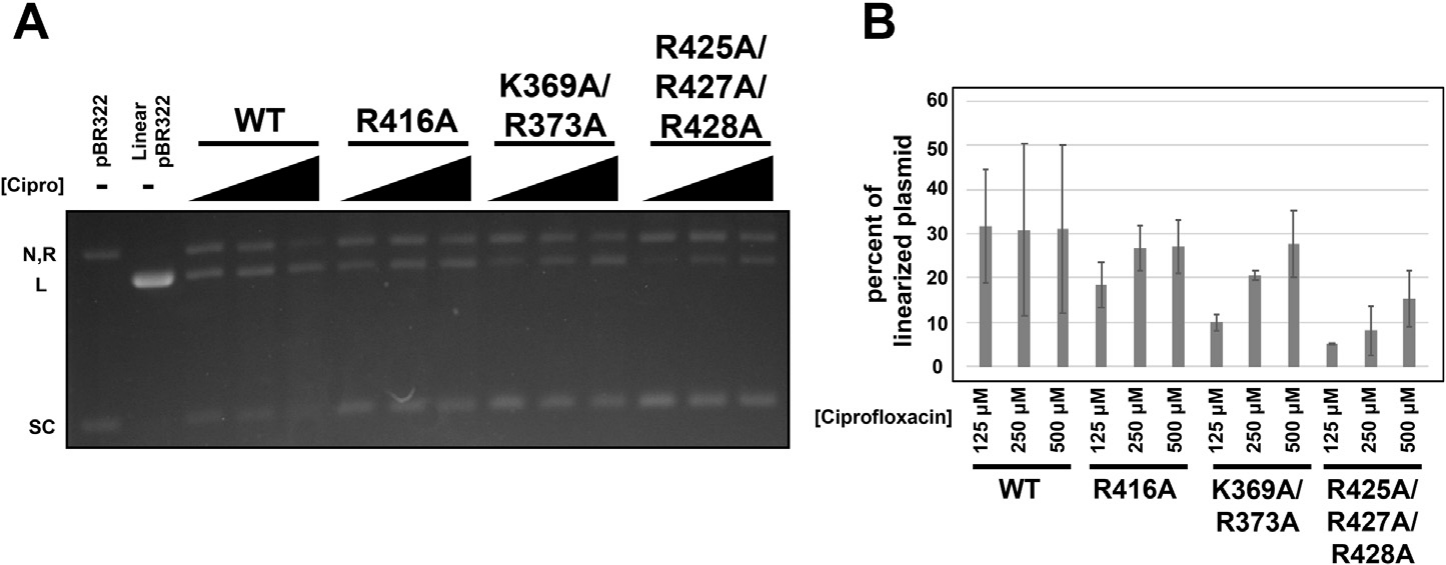
**Cleavage activity of DNA gyrase.***A*, DNA cleavage activity of DNA gyrase formed with the indicated GyrA construct. Shown is a representative ethidium bromide–stained 1% agarose gel. The negative control is pBR322 plasmid DNA, and the linear control is the pBR322 plasmid digested with HindIII. DNA gyrase (250 nM GyrA and 1.2 μM GyrB) was incubated with 7 nM of pBR322 plasmid DNA and increasing amounts of ciprofloxacin (125 μM, 250 μM, and 500 μM). *B*, *bar graph* depicting the average percent of linearized plasmid for each reaction of gyrase formed with the indicated GyrA construct at each ciprofloxacin concentration. Error bars denote the SD from three independent experiments.

As these mutants are located in the C-gate, we hypothesized that the reduced supercoiling activity is the result of disrupted binding between the C-gate and the T-segment. However, supercoiling activity not only requires efficient T-segment exit but also functional cleavage of the G-segment. To determine if the introduced mutations impact G-segment cleavage, we performed DNA cleavage assays on plasmid DNA in the presence of increasing concentrations of ciprofloxacin, a well-characterized gyrase poison. In the presence of 500 μM ciprofloxacin, WT DNA gyrase was able to linearize 30% of the plasmid DNA in the reaction (Fig. 4, *A* and *B*). DNA gyrase mutants harboring either GyrA–R416A or GyrA–K369A/R373A were also able to cleave DNA to a similar extent as WT, with 29% and 27% of the plasmid DNA in the reaction being linearized, respectively (Fig. 4, *A* and *B*). DNA gyrase reconstituted with GyrA–R425A/R427A/R428A was still active in cleavage but did not form as much linearized DNA as WT protein; only 15% of the plasmid was linearized in the presence of 500 μM ciprofloxacin (Fig. 4, *A* and *B*). Interestingly, all the DNA gyrase complexes reconstituted with mutant GyrA displayed a dose–response behavior in the presence of increasing amounts of ciprofloxacin, but this trend was not observed with the complex formed with WT GyrA. Instead, the WT protein complex forms the same amount of linear plasmid with each ciprofloxacin concentration; a trend has been observed in other studies that have performed cleavage assays with similar concentrations of ciprofloxacin (27, 28). Because the DNA gyrase complex harboring the GyrA–R425A/R427A/R428A mutation was still able to cleave the G-segment, we performed cleavage assays with gyrase containing GyrA–R425A/R427A/R428A for longer periods of time and with higher amount of ciprofloxacin. Reactions that were allowed to proceed for longer periods of time or with more ciprofloxacin produced more linear plasmid (22–28%) and approached values closer to that of the WT protein (Fig. S4). Together, these data show that gyrase-containing mutant GyrA constructs are indeed still able to cleave DNA.

### DNA-stimulated ATPase activity is preserved in gyrase formed with C-gate mutants

**Figure 5:**
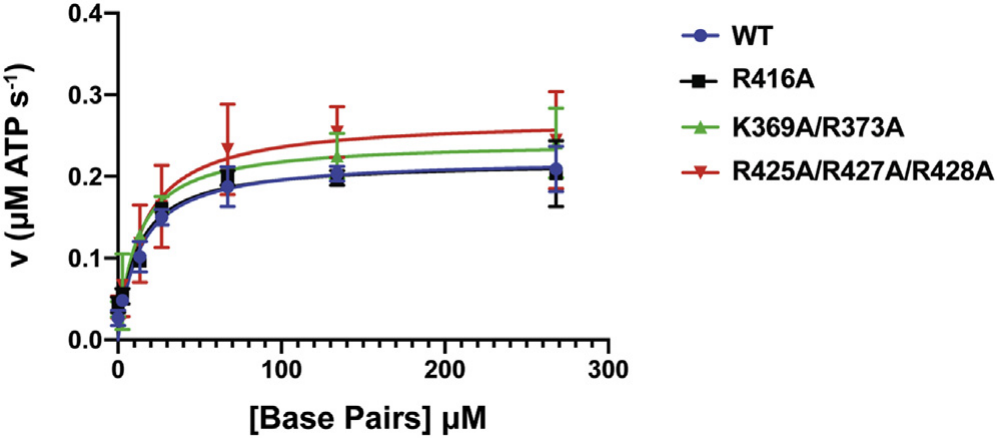
**DNA-stimulated ATPase activity of DNA gyrase.** Graph showing the reaction velocity in micromolar ATP s^−1^ on the y-axis and concentration of DNA base pairs in micromolar on the x-axis (0–268 μM base pairs or 0–100 nM pUC19 plasmid). The data for DNA gyrase formed with WT GyrA are shown in *blue*, the GyrA–R416A mutant in *black*, the GyrA–K369A/R373A mutant in *green*, and the GyrA–R425A/R427A/R428A mutant in *red*. The final concentrations of GyrA and GyrB used in this experiment are 500 nM and 600 nM, respectively. Error bars denote the SD from 3 to 6 independent experiments.

Defects in supercoiling activity can be attributed to reduced ATPase activity as well as defects in T-segment passage through the enzyme. To determine if the loss of supercoiling activity in the mutants is a result of reduced DNA-stimulated ATPase activity instead of reduced T-segment transport through the C-gate, a coupled ATPase activity assay was performed in the presence of plasmid DNA (Fig. 5). WT DNA gyrase catalyzes ATP hydrolysis with a *k*_cat_ of 0.37 (±0.02) s^−1^ and has a *K*_m, DNA_ of 13.3 (±2.2) μM base pairs (4.9 nM plasmid) (Table 1) (27). Gyrase mutants formed with either GyrA–R416A or GyrA–K369A/R373A were able to hydrolyze ATP at a similar rate to that of WT. GyrA–R416 hydrolyzed ATP with a *k*_cat_ of 0.36 (±0.02) s^−1^ and GyrA–K369A/R373A with a *k*_cat_ of 0.41 (±0.02) s^−1^ (Table 1). Importantly, reconstituted gyrase harboring the triple mutant GyrA–R425A/R427A/R428A hydrolyzed ATP at a slightly higher, but comparable rate as the other constructs, with a *k*_cat_ of 0.45 (±0.03) s^−1^ (Table 1). All mutant constructs gave *K*_m, DNA_ values close to that of WT, with the GyrA–R416A k_m, DNA_ of 11.8 (±2.3) μM base pairs (4.4 nM plasmid), the GyrA–K369A/R373A k_m, DNA_ of 11.5 (±2.7) μM base pairs (4.3 nM plasmid), and the GyrA–R425A/R427A/R428A *K*_m, DNA_ of 14.8 (±4) μM base pairs (5.5 nM plasmid), illustrating that the DNA substrate had a similar affinity to gyrases formed with the mutant GyrA constructs as the WT protein complex. These data support the conclusion that the defects in supercoiling activity observed in the GyrA–K369A/R373A and GyrA–R425A/R427A/R428A mutants are not due to a defect in ATP hydrolysis or a decreased affinity of the enzyme for DNA.

**Table 1:** DNA-stimulated ATPase activity of DNA gyrase

GyrA Construct	WT	R416A	K369A/R373A	R425A/R427A/R428A
V_max_	0.22 ± 0.01	0.22 ± 0.01	0.24 ± 0.01	0.27 ± 0.02
*k*_0_ (s^−1^)	0.028 ± 0.009	0.043 ± 0.009	0.038 ± 0.009	0.04 ± 0.013
*k*_cat_ (s^−1^)	0.37 ± 0.02	0.36 ± 0.02	0.41 ± 0.02	0.45 ± 0.03
*K*_m, DNA_ (μM)	13.3 ± 2.2	11.8 ± 2.3	11.5 ± 2.7	14.8 ± 4

The ATPase activity data were fit to the Michaelis–Menten equation, and the different parameters were extracted. The error is the SD from the fit. *k*_0_ is calculated from reactions without plasmid DNA added. The error in *k*_0_ is the SD from three independent experiments.

## Discussion

An impressive amount of molecular detail is known about the ATP-dependent two-gate strand passage mechanism of DNA gyrase. However, many questions remain about how the enzyme coordinates T-segment movement from the cavity formed between the N-gate and DNA gate, through the cleaved G-segment, and into the cavity formed between the DNA gate and C-gate. To understand better the latter step of T-segment transport in type II topoisomerases, we engineered alanine mutations in conserved basic residues in the C-gate of the A subunit of *S. pneumoniae* DNA gyrase. Alanine mutations of K369 and R373 had a minor effect on the supercoiling activity of gyrase, whereas a triple mutant, R425A/R427A/R428A, had a marked effect on the supercoiling activity of the enzyme. These data, along with the structure of *S. pneumoniae* DNA gyrase that places R425, R427, and R428 in the C-gate, suggest that these conserved residues are important for T-segment transport (20, 26).

To determine if these mutations affected other steps in the mechanism of the enzyme, both DNA-dependent ATPase and DNA cleavage assays were performed. None of the mutations had any effect on the enzyme ability to hydrolyze ATP in the presence of DNA. Surprisingly, compared with the WT and other mutants, the R425A/R427A/R428A mutant needed longer times to produce similar amounts of cleaved DNA. Given the distance of these residues from the active site and the evidence from CD spectra suggesting that the overall fold of the enzyme is still intact, it is unlikely that mutating these residues has a direct effect on the structure of the active site. However, it is still possible that part of the defect in supercoiling observed could be due to decreased cleavage complex formation.

The crystal structure of *S. pneumoniae* GyrA shows that R425, R427, and R428 are found at the GyrA dimer interface that forms the C-gate (Fig. 1) (20, 26). Thus, six basic residues, three from each of the two monomers that make up the dimer, are located directly at the surface of the cavity and by the C-gate. This creates a large positively charged patch of basic amino acids that is poised to interact with the T-segment as it traverses through the enzyme. Importantly, this basic patch is conserved in gyrases from other bacteria as well. Inspection of the high-resolution 2.3 Å crystal structure of *Staphylococcus aureus* gyrase depicts three residues (R429, R431, and R432) forming a similar patch at the C-gate (29). Similar patches are also found in the structures of gyrase from *Escherichia coli* and *Bacillus subtilis*, highlighting a possible conserved function of these residues in T-segment passage (30, 31). The importance of this basic patch along with K369 and R373 is highlighted by the cryo-EM structure of *S. pneumoniae* GyrA (20). This structure shows a T-segment 4.8 to 6.7 Å away from these residues, illustrating their interaction with the DNA at the C-gate. Taking our data along with this structural information, we propose a model where these residues are able to help coordinate T-segment motion toward the C-gate and provide a basic patch to interact with it at the C-gate. This interaction may communicate the closing of the DNA gate and opening of the C gate as the T-segment passes through one and interacts with the other.

Important information about C-gate opening has been provided by the crystal structures of *S. cerevisiae* topoisomerase II (10, 32). These structures are the only depictions of a type II topoisomerase with the C-gate in both the open and closed conformations (10, 32). In the structure with a closed C-gate, *S. cerevisiae* topoisomerase II has four basic residues (K1062, K1065, R1120, and R1128) at the dimer interface pointing up into the cavity where a T-segment would be (32). Similarly to the residues found in DNA gyrase, these residues could play a role in T-segment passage. However, these residues span a longer distance than that of the basic residues found in DNA gyrase. In *S. cerevisiae* topoisomerase II, the side chains are 11 Å apart at their closest and are 29 Å apart at their furthest. The crystal structure of the core cleavage complex of *S. cerevisiae* topoisomerase II with an open C-gate depicts some rearrangement of these basic residues with N1042, R1045, K1062, and R1120 at the now-separated dimer interface, while the other basic residues (K1065 and R1128) are positioned away from where the T-segment is predicted to exit (10). The observed conformational changes could help facilitate the movement of the T-segment out of the enzyme.

A structure of a bacterial gyrase with an open C-gate will be necessary to determine if the basic residues identified here undergo similar conformational changes. We can predict that as the dimeric interface of the C-gate separates, there will be some change in the orientation of the residues at the gate, similar to what is observed in the crystal structure with an open C-gate from *S. cerevisiae*. Changes in the orientation of these residues may help guide the T-segment out of the enzyme. To understand these steps better, it is necessary to obtain additional high-resolution structures of type II topoisomerases with a T-segment bound.

Taking our data, along with information from both cryo-EM and crystal structures, we propose a model for T-segment passage that utilizes the basic patch identified here. The T-segment must pass through the DNA gate and into the cavity formed by the coil-coiled domain of GyrA to exit the enzyme. We propose that the basic residues found at the bottom of the C-gate can act as a large basic patch to attract the DNA duplex as it enters this cavity. After the T-segment passes through the WHDs, we predict that this basic patch creates a positively charged electrostatic area that guides the T-segment toward the C-gate and provides a stable interaction site until the DNA duplex can exit the enzyme. When the T-segment interacts with this basic patch, it may also act as a communication signal between the C-gate and DNA gate by inducing a conformational change that could trigger the DNA gate to close. Ultimately, the C-gate itself must undergo conformational changes to usher the T-segment out of the enzyme. Given the conformational changes that occur in the opened and closed C-gates illustrated by the crystal structure of *S. cerevisiae* topoisomerase II, we predict that the C-gate of DNA gyrase will open in a similar manner. During this opening, changes in the orientations of the basic residues could guide the T-segment out of the enzyme. Overall, our data point to a model whereby the basic residues identified in our focused mutagenesis screen support T-segment passage and stability at the C-gate.

## Experimental procedures

### Plasmid constructs

*S*. *pneumoniae gyrA* and *gyrB* were cloned into the pMCSG7 vector as described previously (20, 24). Mutations were introduced into *gyrA* in pMCSG7 with complementary mutagenic primers (Integrated DNA Technologies). Phusion polymerase (New England Biolabs) was used for site-directed mutagenesis with the manufacturer’s suggested protocol with one exception, the extension time used for the PCR was 17 min. Sanger sequencing was performed on mutated plasmids to determine both if the intended mutations were introduced into the plasmid and any unintended mutations were not introduced.

### Protein purification

Constructs of gyrA and gyrB were expressed and purified as described previously (20). Briefly, *E. coli* BL21-DE3 cells were grown at 37 °C until they reached an *A*_600_ of 0.8 to 1.0. Upon reaching this optical density, the cells were induced with 500 mM IPTG for 14 to 16 h at 16 °C. Cells were then spun down at 4000 rpm in an F8S-6x1000y fixed angle rotor (Thermo Fisher) in a Thermo Scientific Sorvall Evolution centrifuge (Thermo Scientific) and resuspended in the binding buffer (50 mM Tris HCl, pH 8, 300 mM NaCl, 5 mM imidazole, 5% glycerol). After resuspension, cells were lysed by sonication, and the lysate was clarified by centrifugation at 38,000 rpm in a Ti-70 rotor (Beckman Coulter) in an Optima XE-90 ultracentrifuge (Beckman Coulter). The clarified lysate was passed over a Nickel-Sepharose (Ni Sepharose 6 fast flow, GE Healthcare) column that was equilibrated with the binding buffer. After the lysate was passed over the column, three washes were performed. The first with five column volumes of the binding buffer, the second with five column volumes of wash buffer I (50 mM Tris HCl, pH 8, 300 mM NaCl, 20 mM imidazole, 5% glycerol), the third with five column volumes of wash buffer II (50 mM Tris HCl, pH 8, 300 mM NaCl, 35 mM imidazole, 5% glycerol). Gyrase A was eluted with five column volumes of the elution buffer (50 mM Tris HCl, pH 8, 300 mM NaCl, 300 mM imidazole, 5% glycerol). Fractions eluted from the Ni-Sepharose beads were analyzed by Coomassie-stained SDS-PAGE gels. Fractions containing GyrA were then pooled and dialyzed into heparin buffer A (50 mM Tris HCl, pH 8, 150 mM NaCl, 0.5 mM EDTA, 1 mM DTT) at 4 °C overnight. Dialyzed fractions were passed over a heparin column (HiTrap Heparin High Performance, GE Healthcare) with a gradient from 0% to 100% heparin buffer B (50 mM Tris HCl, pH 8, 300 mM NaCl, 300 mM imidazole, 5% glycerol). Eluted fractions were again analyzed by Coomassie-stained SDS-PAGE gels. Fractions containing protein were dialyzed into a high-salt S300 buffer (50 mM Tris HCl, pH 8, 600 mM KCl, 1 mM EDTA) at 4 °C overnight. After dialysis, the protein was passed over an S-300 (Sephacryl S-300 HR, HiPrep 16/60, GE Healthcare) column. Peak fractions were analyzed by Coomassie-stained SDS-PAGE gels to determine purity. Pure fractions were then pooled, concentrated, and stored at −80 °C. GyrB was purified by the same method with one major difference. The fractions eluted from the heparin column were dialyzed into a low-salt S300 buffer (50 mM Tris HCl, pH 8, 150 mM KCl, 1 mM EDTA), and this same buffer was used as the eluent during gel filtration on the S-300 column.

### CD

Purified GyrA proteins were diluted to 10 μM in S300 buffer (50 mM Tris HCl, pH 8.0, 600 mM KCl, 1 mM EDTA). Protein concentrations were checked by taking the absorbance at 280 nm with a Nanodrop spectrophotometer (ND-1000). Samples were placed in a 180 μl cuvette (Precision Cells Inc) with a path length of 0.5 mm. Spectra were obtained with a Jasco CD spectrophotometer (J-815) equipped with a temperature-controlled sample holder (Peltier PFD-425S) that was kept at 25 °C. Jasco spectra analysis tools software was used to convert ellipticity to mean residue molar ellipticity.

### DNA gyrase supercoiling assay

Before performing the supercoiling reaction, either 35 nM of pBR322 plasmid DNA or pUC19 plasmid DNA was relaxed in a 10-μl reaction with 430 nM *E. coli* topoisomerase I in the relaxation buffer (10 mM Tris, pH 8.0, 40 mM KCl, 5 mM MgCl_2_). These reactions proceeded for 1 h at 25 °C. After 1 h, topoisomerase I was heat-inactivated by incubating the mixture at 70 °C for 15 min. Before each 35-μl supercoiling reaction, GyrA and GyrB were diluted with the gyrase dilution buffer (50 mM Tris HCl, pH 7.5, 0.2 M KCl, 5 mM DTT, 1 mM EDTA, 3 mg/ml bovine serum albumin (BSA), 50% glycerol). GyrA and GyrB were mixed and allowed to incubate on ice for 10 min. During this incubation period, a master mix of relaxed DNA (10 nM final concentration), 3X gyrase cleavage reaction buffer (105 mM Tris HCl, pH 7.5, 18 mM MgCl_2_, 5.4 mM spermidine, 72 mM KCl, 15 mM DTT, 1.08 mg/ml BSA, 19.5% glycerol (w/v)), and water was made. This was added to the incubated GyrA and GyrB mixture. The final concentrations of GyrA and GyrB used in the reactions were 400 nM GyrA:800 nM GyrB, 200 nM GyrA:400 nM GyrB, and 100 nM GyrA:200 nM GyrB. Relaxed pBR322 plasmid DNA was added at a final concentration of 10 nM. The zero time point depicted on the gels comes before the addition of ATP to each reaction mixture. To initiate each reaction, ATP was added to a final concentration of 1.5 mM. Reactions performed with pUC19 were done as above, but the reactions were carried out with 5 nM plasmid, 250 nM GyrB, and 125 nM GyrA in 75 mM Tris HCl, pH 7.5, 7.5 mM MgCl_2_, 7.5 mM DTT, 0.0075 mg/ml BSA, 30 mM KCl, and 500 mM potassium glutamate. All supercoiling reactions were stopped at the indicated time points by adding 1 μl of 500 mM EDTA and 2 μl of 10% SDS. Supercoiling reaction products were electrophoresed in a 1% agarose gel run at 20 V for 16 h. DNA bands were visualized by staining with ethidium bromide and imaged on an ImageQuant LAS 4010 imaging system. Quantitation was performed using ImageQuant (version 5.2). Background correction was done using the rolling-ball method. To determine the percent of pBR322 plasmid that was supercoiled, for each lane, a large box was drawn to encompass the nicked and relaxed topoisomer band along with the other relaxed topoisomer species found and the values of the pixels within the box added. Next, a small box was drawn around the band that corresponds to supercoiled plasmid and the values of the pixels within the box added. The sum of the values from these boxes corresponds to the total amount of DNA in each lane (total lane count). To determine the amount of supercoiled plasmid (supercoiled count), for each lane, the supercoiled count was divided by the total lane count corresponding to that lane. This value was multiplied by 100 to give a percentage of total pBR322 DNA that was supercoiled.

### DNA gyrase cleavage assay

DNA cleavage assays were performed as previously described by Fisher *et al.* (28). A 20-μl cleavage reaction was performed by incubating each GyrA construct with WT GyrB for 10 min on ice. Both GyrA and GyrB were diluted with a gyrase dilution buffer (50 mM Tris HCl, pH 7.5, 0.2 M KCl, 5 mM DTT, 1 mM EDTA, 3 mg/ml BSA, 50% glycerol) before incubation. The final concentration of GyrA in each 20 μl reaction is 250 nM, and the final concentration of GyrB is 1.2 μM in each reaction. During incubation, separate tubes were prepared either without inhibitor or with ciprofloxacin (Sigma, 17850-5G-F) to give final ciprofloxacin concentrations of 0 μM, 125 μM, 250 μM, and 500 μM. To these tubes, pBR322 plasmid DNA was added to a final concentration of 7 nM, 6.7 μl of 3X gyrase cleavage reaction buffer (105 mM Tris HCl, pH 7.5, 18 mM MgCl_2_, 5.4 mM spermidine, 72 mM KCl, 15 mM DTT, 1.08 mg/ml BSA, 19.5% glycerol (w/v)) and water were added to give a final reaction volume of 20 μl. After GyrA and GyrB were incubated on ice for 10 min, they were added to the above reaction mixture. The reaction proceeded at 25 °C for 1 h. After 1 h, 3 μl of 2% SDS was added and the reaction was vortexed briefly. Next, 3 μl of 1 mg/ml proteinase K (Sigma P2308) was added and again the mixture was briefly vortexed before being allowed to incubate for 30 min at 37 °C. To obtain a linear pBR322 plasmid to use as a control, 400 ng of plasmid was incubated with HindIII (New England Biolabs) following the manufacturer’s suggested protocols. To determine the amount of cleavage complex formed in each reaction, samples were mixed with the gel loading dye and separated on a 1% agarose gel that was run at 15 V for approximately 14 h. DNA bands were observed by staining with ethidium bromide and visualized on an ImageQuant LAS 4010 imaging system. Quantitation was performed using ImageQuant (version 5.2). Background correction was done by the rolling-ball method in ImageQuant. To determine the percent of pBR322 plasmid that was linearized (cleaved by DNA gyrase) in each reaction, we first drew a box around the top band of each lane that corresponds to a mixed population of relaxed and nicked DNA in the sample. We then drew separate boxes around the band that corresponds to the linearized plasmid (middle band) and supercoiled band (bottom band). Calculations were performed by first summing the values obtained for species described above to determine the total amount of DNA present in each lane. To obtain a percentage of linearized plasmid in each lane, we divided the value obtained for the linearized band (middle band) by the total lane counts and multiplied the fraction by 100.

### ATPase assay

ATPase assays were performed based on previously published protocols (27, 33). These protocols use an assay in which ATP hydrolysis is coupled to reduced NADH oxidation. First, 500 nM of GyrA and 600 nM of GyrB were incubated on ice for at least 10 min. Next, a mixture of the indicated concentration of pUC19 plasmid DNA (0–100 nM pUC19 plasmid or 0–268 μM base pairs) in 75 mM Hepes, pH 7.0, 7.5 mM MgCl_2_, 0.075 mg/ml BSA, 30 mM KCl, 7.5 mM DTT, 375 mM potassium glutamate, 2 mM ATP, 6 U pyruvate kinase, and 8 U lactate dehydrogenase was made. The reaction was initiated by adding the incubated protein complex and a mixture of 2 mM phosphoenolpyruvate and 0.16 mM NADH dissolved in 50 mM Tris, pH 8.0, to the DNA-containing mixture. Immediately after addition of the protein and NADH, the absorbance at 340 nM was measured in a UV/VIS spectrophotometer with a path length of 1 cm (HP8452, Agilent Technologies) every 5 s for 10 min. The linear portion of the resulting spectra was analyzed by a least squares fit method. The results were then plotted, and the data were analyzed using Prism (GraphPad) and Origin (OriginLab) to fit a Michaelis–Menten formula to obtain *V*_max_, *K*_m_, and *k*_cat_. The value for *k*_0_ was determined experimentally by performing reactions without DNA present.

## Data availability

All data that are part of this study are included within this article.

## Supporting information

This article contains supporting information.

## Conflict of interest

The authors declare that they have no conflicts of interest with the contents of this article.
