# Supplementary material for: Basic residues at the C-gate of DNA gyrase are involved in DNA supercoiling
Source: J Biol Chem. 2021 Jul 22;297(2):101000. doi: 10.1016/j.jbc.2021.101000 (PMC8368997; doi:10.1016/j.jbc.2021.101000)
Supplement: Supplemental Figures S1–S4 [file mmc1.pdf]

# Supporting Information

# Basic residues at the C-gate of DNA gyrase are involved in DNA supercoiling.

Eric M. Smith and Alfonso Mondragón*

Department of Molecular Biosciences, Northwestern University, Evanston Illinois 60208

Supporting Figures S1-S4.

**A**

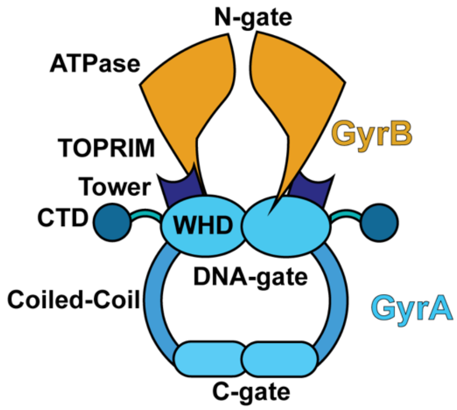


**B**

```
                 350       360       370       380       390       400
                  |         |         |         |         |         |
S.pnuemoniae    LDAYIEHQKEVVVRRTRFDKEKAEARAHILEGLLIALDHIDEVIRIIRASETDAEAQAEL    407
E.coli          IAAFVRHRREVVTRRTIFELRKARDRAHILEALAVALANIDPIIELIRHAPTPAEAKTAL    410
S. enterica     ISAFVRHRREVVTRRTIFELRKARDRAHILEALAIALANIDPIIELIRRAPTPAEAKAAL    410
B. subtilis     LEHYLDHQKVVIRRRTAYELRKAEARAHILEGLRVALDHLDAVISLIRNSQTAEIARTGL    418
S. aureus       LYHYLEHQKDVVTRRTKYNLKKAQDRAHILEGLRIALDHIDEIITLIRESQSDSEAMEGL    411
                :  :: *:: *: *** :: .**. ******.* :** ::* :* :** : :   *   *

                                                   410       420       430
                                                    |         |         |
S.pnuemoniae    M----------------------------------SKFKLSERQSQAILDMRLRRLTGLE    433
E.coli          VANPWQLGNVAAMLERAGDDAARPEWLEPEFGVRDGLYYLTEQQAQAILDLRLQKLTGLE    470
S. enterica     ISRPWDLGNVAAMLERAGDDAARPEWLEPEFGVRDGQYYLTEQQAQAILDLRLQKLTGLE    470
B. subtilis     I----------------------------------EQFSLTEKQAQAILDMRLQRLTGLE    444
S. aureus       Q----------------------------------NRFNLSERQAQAILDMRLRRLTGLE    437
                                                     : *:*:*:*****:**::*****
```

**Figure S1. (A)** Cartoon depicting the overall architecture of the DNA gyrase $A_2B_2$ heterotetramer. Domains of GyrA are represented in shades of blue whereas domains of GyrB are represented in orange. All gates are shown in a closed conformation. **(B)** Sequence alignment of residues 358-443 of *S. pneumoniae* GyrA and GyrA from other organisms (*Escherichia coli*, *Salmonella enterica*, *Bacillus Subtilis*, and *Staphylococcus aureus*) performed with Clustal Omega (EMBL-EBI). Residue numbers shown above the sequences correspond to *S. pneumoniae* GyrA.

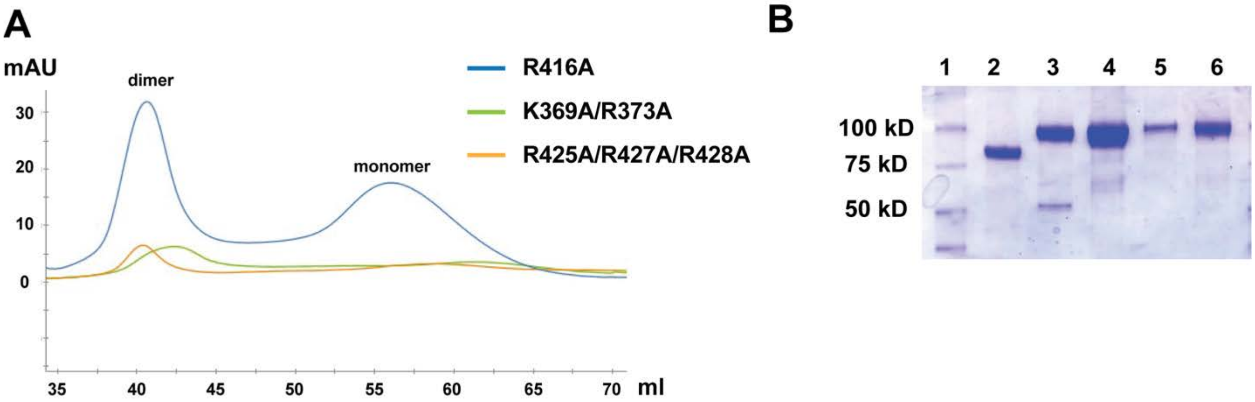


**Figure S2. (A)** Overlayed chromatograms from passing each mutant GyrA construct over a Sephacryl S-300 HR HiPrep 16/60 gel filtration column. Peaks corresponding to the dimeric and monomeric species of GyrA found in each purification are marked on the chromatogram. **(B)** Coomassie stained SDS-PAGE gel showing bands corresponding to each GyrB and GyrA protein construct used in this study. Lane 1 is a protein ladder, Lane 2 is purified GyrB, Lane 3 is purified WT GyrA, Lane 4 is purified GyrA-R416A, Lane 5 is purified GyrA-K369A/R373A, and Lane 6 is purified GyrA-R425A/R427A/R428A.

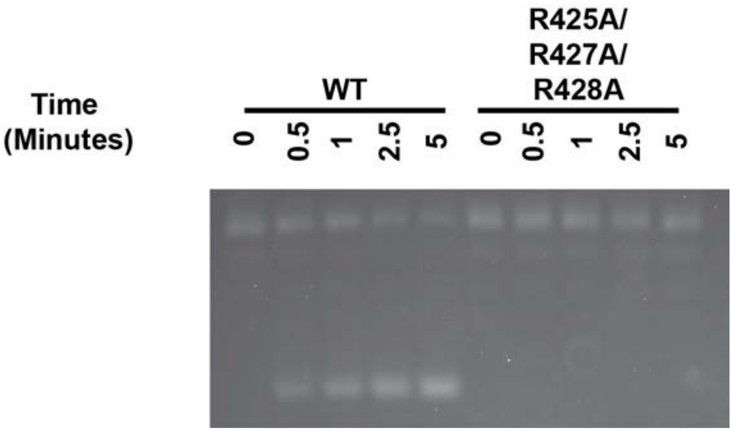


**Figure S3.** 1% agarose gel stained with ethidium bromide depicting the results of a supercoiling assay of DNA gyrase reconstituted with 125 nM of the indicated construct of GyrA and 250 nM WT GyrB with 5 nM relaxed pUC19 plasmid DNA. The four time points (in minutes) are counted after ATP addition to each reaction.

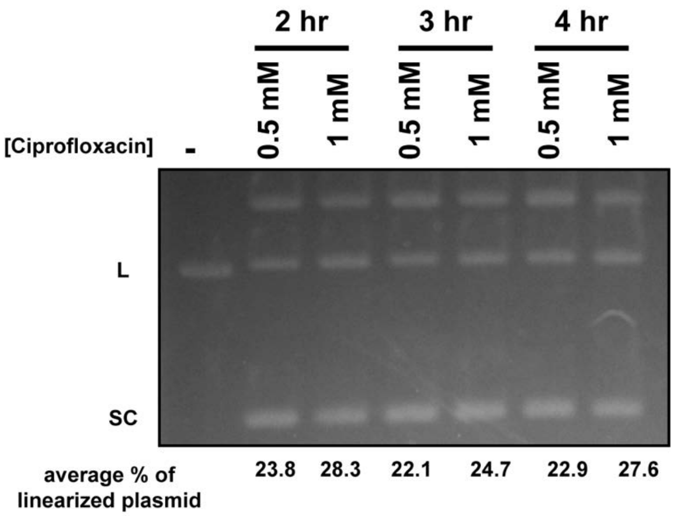


**Figure S4.** DNA cleavage activity of gyrase formed with 250 nM GyrA-R425A/R427A/R428A mutant and 1.2 μM GyrB. Shown is a 1% agarose gel stained with ethidium bromide. Each cleavage assay was carried out in the presence of either 500 μM or 1 mM of ciprofloxacin for the indicated amount of time. The final concentration of pBR322 used in each reaction was 7 nM. The average percent of linearized DNA is calculated from three independent experiments.
